# GDmicro: classifying host disease status with GCN and deep adaptation network based on the human gut microbiome data

**DOI:** 10.1093/bioinformatics/btad747

**Published:** 2023-12-12

**Authors:** Herui Liao, Jiayu Shang, Yanni Sun

**Affiliations:** Department of Electrical Engineering, City University of Hong Kong, Kowloon, Hong Kong (SAR), 518057, China; Department of Electrical Engineering, City University of Hong Kong, Kowloon, Hong Kong (SAR), 518057, China; Department of Electrical Engineering, City University of Hong Kong, Kowloon, Hong Kong (SAR), 518057, China

## Abstract

**Motivation:**

With advances in metagenomic sequencing technologies, there are accumulating studies revealing the associations between the human gut microbiome and some human diseases. These associations shed light on using gut microbiome data to distinguish case and control samples of a specific disease, which is also called host disease status classification. Importantly, using learning-based models to distinguish the disease and control samples is expected to identify important biomarkers more accurately than abundance-based statistical analysis. However, available tools have not fully addressed two challenges associated with this task: limited labeled microbiome data and decreased accuracy in cross-studies. The confounding factors, such as the diet, technical biases in sample collection/sequencing across different studies/cohorts often jeopardize the generalization of the learning model.

**Results:**

To address these challenges, we develop a new tool GDmicro, which combines semi-supervised learning and domain adaptation to achieve a more generalized model using limited labeled samples. We evaluated GDmicro on human gut microbiome data from 11 cohorts covering 5 different diseases. The results show that GDmicro has better performance and robustness than state-of-the-art tools. In particular, it improves the AUC from 0.783 to 0.949 in identifying inflammatory bowel disease. Furthermore, GDmicro can identify potential biomarkers with greater accuracy than abundance-based statistical analysis methods. It also reveals the contribution of these biomarkers to the host’s disease status.

**Availability and implementation:**

https://github.com/liaoherui/GDmicro.

## 1 Introduction 

In recent years, many studies have shown strong associations between the human gut microbiome and several human diseases (Yu *et al.* 2017, Kwong *et al.* 2018, Gomaa 2020). For example, a meta-analysis of large-scale metagenomic samples shows that dozens of specific bacteria are enriched in colorectal cancer (CRC) patients across different countries (Wirbel *et al.* 2019). Another microbiome-related study found a reduced complexity of the bacterial phylum *Firmicutes* in inflammatory bowel disease (IBD) patients (Manichanh *et al.* 2006). With the in-depth study of *Firmicutes*, anti-inflammatory properties of many species under this phylum have been revealed, implying their potential utilities in promoting gut health. These observations indicate that the composition of gut microbes may provide important information for distinguishing case and control samples of a particular disease. Given the accumulating evidence on the associations between diseases and the human gut microbiome, there is a need to develop a more accurate microbiome-based host disease status classification model. Such a model has the potential to identify more informative biomarkers for downstream analysis than abundance-based statistical analysis (Curry *et al.* 2021). The goal of this study is to create a more accurate host disease status classification model using data from the human gut microbiome and incorporating advanced functions for biomarker discovery.

Many computational methods have been developed to classify host disease status based on the human gut microbiome data. The abundance of gut microbes is a major feature used by these tools (Curry *et al.* 2021). Given the gut microbial composition abundance data, these methods apply traditional machine learning or deep-learning methods to distinguish case and control samples of a specific disease. The tools utilizing traditional machine learning include MetAML (Pasolli *et al.* 2016) and SIAMCAT (Wirbel *et al.* 2021). MetAML takes gut microbial compositional data as input and then applies random forest and support vector machines to classify host disease status. To improve the classification performance, it combines *k*-fold cross-validation and the grid search strategy to tune the best hyperparameters for models. SIAMCAT (Wirbel *et al.* 2021) is a toolbox that relies on various regression models, such as ridge regression, to classify host disease status using gut microbial compositional data.

In addition to traditional machine-learning methods, deep-learning models have shown promising results in classifying host disease status (Oh and Zhang 2020, Reiman *et al.* 2020). One relevant work is DeepMicro (Oh and Zhang 2020), which applies an autoencoder model to learn the deep representation of input gut microbial compositional features. Then, the multi-layer perceptron model is employed to classify host disease status with the learned latent features. PopPhy-CNN (Reiman *et al.* 2020) is another popular deep-learning-based tool. It takes gut microbial compositional data and a phylogenetic tree as input and utilizes a novel convolutional neural network learning framework for host disease status classification.

Despite the promising results, existing tools have not addressed two challenges well. The first challenge is the limited number of labeled data. Although there has been rapid growth in microbiome data collection, a large number of samples still lack detailed metadata annotations. According to a recent study, only 7.8% of the 444 829 human microbiome samples had explicit information on host disease status (Abdill *et al.* 2022), partly because of the high cost of obtaining labels for microbiome data (Shen *et al.* 2022). As a result, limited labeled data pose challenges for most learning models. Second, many available methods ignore the domain discrepancy problem. For example, in our *k*-fold cross-validation experiment on CRC, DeepMicro achieves a 0.803 area under the curve (AUC) on the CRC-FR dataset. However, when applied to CRC-US dataset in the cross-study experiment, its performance drops to 0.609. Specifically, data from different studies have many differences due to confounding factors, such as region, ethnicity, and diet, which can lead to changes in the gut microbiome (He *et al.* 2018, Wirbel *et al.* 2019). Thus, the domain difference between training and test data can greatly affect the robustness of the classification model, ultimately leading to poor performance in real applications.

### 1.1 Overview of our method

**Figure 1.**
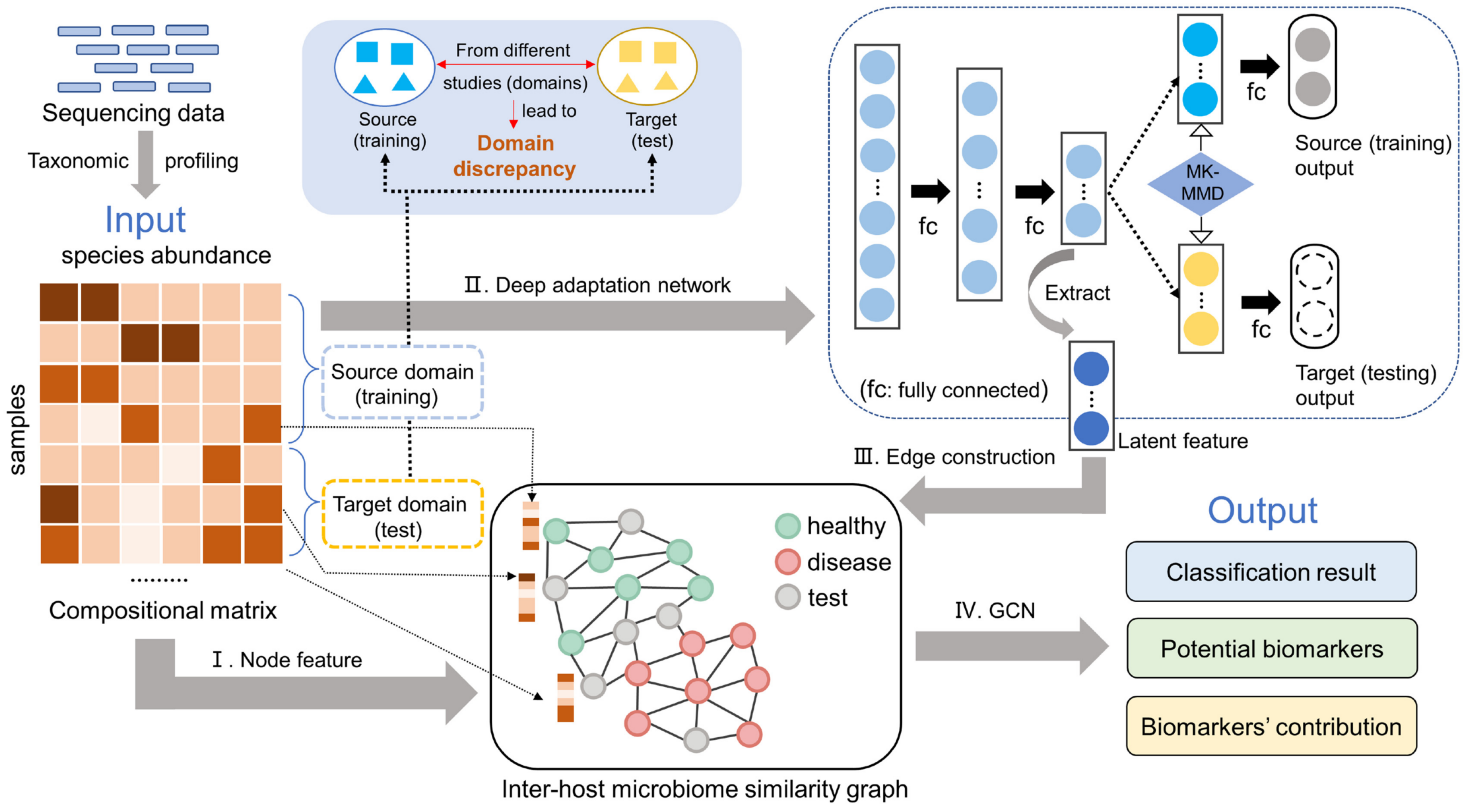
The overall workflow of GDmicro. (I) Representing nodes with species abundance vectors in the inter-host microbiome similarity graph. (II) Applying the deep adaptation network to learn transferable latent features. The deep adaptation network reduces the domain discrepancy between training and test data. (III) Constructing edges of the inter-host microbiome similarity graph with the Euclidean distance of learned latent features from compositional data. (IV) Training a GCN model to classify disease status for test samples. It is worth noting that the domain adaptation and GCN models were initially developed by Long *et al.* (2015) and Kipf and Welling (2017), respectively. In our method, we have employed and further optimized these models to achieve better performance for our problem.

Given the limited labeled data and the rapid accumulation of unlabeled data, we formulate the host disease status classification problem as a semi-supervised learning task, which uses both labeled and unlabeled data for feature learning (van Engelen and Hoos 2020). It should be noted that, to prevent test data leakage, classical semi-supervised learning methods, such as the graph convolutional network (GCN), only utilize the labeled training samples to optimize the loss function in the training process (Zhu *et al.* 2003, Kipf and Welling 2017, Zhou *et al.* 2020). Consequently, these methods exhibit improved performance while effectively avoiding any data leakage concerns in many tasks, including text classification (Yao *et al.* 2019), image classification (Gao *et al.* 2021), and protein function prediction (Gligorijević *et al.* 2021). Here, we present GDmicro, a GCN-based model to utilize the information from both labeled and unlabeled samples for learning and classification. First, an inter-host microbiome similarity graph is built using the gut microbial compositional data. In our graph, nodes represent the compositional abundance features of the hosts’ microbiome (Fig. 1I), and edges represent the similarity of learned latent features between two hosts’ microbiome (Fig. 1II and III). Then, GCN can take this graph as input and incorporate the structural and node abundance features for disease status classification. Because both labeled and unlabeled samples can be integrated into the graph, graph convolution in GCN can utilize local topology for feature passing between two types of samples. Second, to overcome the domain discrepancy problem, we apply a deep adaptation network to learn transferable latent features from the microbial compositional matrix across different domains (Fig. 1II). We validated GDmicro on 10 cohorts covering 5 diseases and compared GDmicro with alternative tools for disease status classification. The results show that GDmicro has consistently higher AUC than other methods. In addition, GDmicro allows users to detect the most important species to disease status classification and explore how these species affect the hosts’ disease status, which provides important information for biomarker discovery.

## 2 Materials and methods

We choose GCN (Kipf and Welling 2017) as the semi-supervised learning engine for our host disease status classification problem, which uses both labeled and unlabeled data for feature learning. GCN has had several successful applications in identifying gene–gene interaction, disease–drug relationship, and interactions between phages and bacteria (Han *et al.* 2019, Shang *et al.* 2021, Yu *et al.* 2021). In the host disease status classification problem, GCN has two distinct advantages. First, it can utilize information from unlabeled samples during graph convolution. Second, the GCN model systematically integrates information from the nodes and edges, which represent the abundance distribution of species and their similarities between hosts. Then, we apply the deep adaptation network to mitigate the impact of domain-specific confounding factors on feature learning, leading to a more robust graph for microbiome data from different studies.

In the following sections, we will first introduce how we encode and construct the inter-host microbiome similarity graph with the gut microbiome data and deep adaptation network. Then, we will describe the GCN model optimized for disease identification and its application in biomarker discovery.

### 2.1 Inter-host microbiome similarity graph G

Increasing studies show that people with sclerosis, obesity, and IBDs have similar gut microbial compositional abundance (Chen *et al.* 2016, Lloyd-Price *et al.* 2019, Palmas *et al.* 2021). One recent work constructed a patient relationship graph based on the similarity of human multi-omics data for disease diagnosis (Li *et al.* 2022). Inspired by these studies, we construct an inter-host microbiome similarity graph G to incorporate microbial compositional similarity and compositional abundance features. The major components of the graph construction are sketched in Fig. 1I–III. Each node in the graph represents a human microbial sample, and the edges represent the similarity between the microbiome data. The node features are the compositional abundance vectors of the samples. Because the compositional abundance data might contain skewness or bias in some features, we apply log10-transformation and *z*-score normalization on the features rather than using the raw data. Specifically, the mean and standard deviation are calculated using the training data, and these values are subsequently employed to normalize the test data following log10-transformation. This standardized normalization approach is applied to all experiments. As a result, each node is encoded with the normalized species abundance vectors.

As mentioned above, similar gut microbial compositional data can indicate similar human disease status. Thus, we use microbial compositional similarity to define the edge between two nodes. Because there is no clear cutoff to determine whether two samples are significantly similar, we employ the *k*-nearest neighbor algorithm to generate the topological structure. Specifically, a node will be connected with its *k* closest nodes in G. Below we will detail how to compute the compositional similarity between different samples (Fig. 1II and III).

#### 2.1.1 Using deep adaptation network to learn transferable features

To learn the most relevant features for edge construction from different studies, we applied a deep adaptation network (Long *et al.* 2015) to learn transferable latent features from compositional abundance matrices of training and test datasets.

The deep adaptation network is initially designed to solve the domain adaptation problem in the field of image processing (Long *et al.* 2015). The main idea behind this is to utilize the multiple kernel variants of maximum mean discrepancies (MK-MMD) (Gretton *et al.* 2012) to measure the difference between the source and the target domain and minimize the domain discrepancies during training. To apply MK-MMD to microbiome data, we added an MK-MMD-based adaptation regularizer to the loss function of a multi-layer fully connected network (Fig. 1II). Denote *x* as the compositional abundance vector of one sample, *y* as the disease status label of the sample, $D_{s}={\left\{\left(x_{i}^{s},y_{i}^{s}\right)\right\}}_{i=1}^{n_{s}}$ as the source domain with $n_{s}$ labeled samples and $D_{t}={\left\{x_{j}^{t}\right\}}_{j=1}^{n_{t}}$ as the target domain with $n_{t}$ unlabeled samples. Then, the loss function of the network can be defined as:

(1) $$\sum_{i=1}^{n_{s}}L(\theta (x_{i}^{s}),y_{i}^{s})+\lambda d_{k}^{2}(D_{s}^{l^{\prime }},D_{t}^{l^{\prime }}),$$

where *L* is the cross-entropy loss function for the disease status classification task, $\theta (x_{i}^{s})$ is the conditional probability that the fully connected network in Fig. 1III assigns $x_{i}^{s}$ to label $y_{i}^{s}$, λ>0 is a penalty parameter, and $l^{\prime }$ is the hidden layer index where the regularizer is effective. Additional details about the domain adaptation regularizer $d_{k}^{2}$ can be found in Supplementary Section S1.1. As a result, by minimizing the loss function with the MK-MMD-based adaptation regularizer, the model can learn the transferable latent features between data from different domains.

After training the deep adaptation network, we applied the trained model to convert input microbial compositional features to latent features from the fully connected network. The equation for the conversion is listed in Equation (2).

(2) $$h^{\left(l+1\right)}=\mathrm{ReLU}(h^{\left(l\right)}w^{\left(l\right)}+b^{\left(l\right)}),\;l\in \{0,1,2\},$$

where $h^{\left(l\right)}$ is the latent features captured from the *l*th hidden layer of the model, and $h^{\left(0\right)}=x$. $w^{\left(l\right)}$ and $b^{\left(l\right)}$ are the learnable weights and biases of the hidden layers. ReLU is the activation function. Finally, each input sample has corresponding latent features $h^{\left(2\right)}$ that contain robust statistic patterns from compositional abundance data.

#### 2.1.2 Edge construction

Given the latent features of all samples, a sample–sample Euclidean distance matrix can be calculated. This matrix effectively captures the microbial compositional similarity across all samples. Then, we employ the *k*-nearest neighbor algorithm to determine whether two samples have a connection. In our algorithm, we will connect the *k* closest samples for each sample according to the distance matrix.

### 2.2 The GCN model

After constructing the graph, we train a GCN model to classify disease status for input samples. The most important component of GCN is the graph convolution layer, which can take advantage of the topological structure for feature learning. Because both labeled and unlabeled samples are connected in the graph, GCN can also utilize node features from unlabeled nodes. If there are *n* samples in the graph, the graph convolution layer is defined as:

(3) $$H^{\left(l+1\right)}=\mathrm{ReLU}({\tilde{D}}^{-\frac{1}{2}}\tilde{A}{\tilde{D}}^{-\frac{1}{2}}H^{\left(l\right)}W^{\left(l\right)}),$$

(4) $$\;\tilde{A}=A+I_{n},$$

where *A* is the $R^{n\times n}$ adjacency matrix of the inter-host microbiome similarity graph, $I_{n}$ is an $R^{n\times n}$ identity matrix, and *D* is the $R^{n\times n}$ degree matrix of $\tilde{A}$. $H^{\left(l\right)}$ refers to the output of *l*th hidden layer and $H^{\left(0\right)}$ is the node feature matrix. $W^{\left(l\right)}$ represents the weight matrix of the hidden layers. As shown in Equation (3), in each convolution layer, the model considers the 1-hop neighborhood of the nodes to calculate new node features. Then, the successive convolution will be applied in the *l* layers, and thus, the model can learn features based on the topological structure to enlarge the receptive field from unlabeled nodes and make a classification. All the nodes will take part in the convolution as shown in Equation (3). But only the labeled nodes are used in optimizing the loss function, which is a cross-entropy loss function for the disease status classification. This operation ensures that the GCN model does not incorporate the label information of test samples into its training process. Finally, the model will assign disease status to unlabeled samples in the classification step.

### 2.3 Discover biomarkers and analyze their contribution to the host disease status with GDmicro

In microbiome-related studies, biomarkers typically refer to species that are highly correlated with diseases and are often considered signatures of certain health conditions (Wirbel *et al.* 2019). Biomarker discovery is an essential task that can help reveal the underlying biological mechanisms of diseases. However, identifying biomarkers using deep-learning models remains challenging. Although methods, such as the ablation approach, are available for interpreting deep-learning models (Wang *et al.* 2021), they cannot be employed in this study due to computational efficiency and performance issues. Specifically, the sheer number of features (over 800 in CRC datasets) makes these methods computationally expensive. In addition, in our specific case, methods like the ablation approach, which requires the removal of one feature from hundreds, cannot produce noticeable differences to the model, making feature selection highly difficult. To tackle these issues, we propose a straightforward yet highly effective performance-based method that utilizes both the input data and the GCN model to extract significant species. Furthermore, based on the identified biomarkers, we design a function that utilizes the GCN model to analyze the biomarkers’ contributions to the host disease status. We will discuss these two methods in the following paragraphs.

First, we utilize the node features in the GCN model for biomarker discovery. Once the graph is given, the performance of the GCN can vary when feeding it with different species. Therefore, we run GDmicro multiple times using a single species as the node feature each time. As a result, each species obtains a corresponding AUC on the training data, allowing for species ranking according to their respective AUCs. Higher-ranked species can be crucial biomarkers for classifying host disease status.

**Figure 2.**
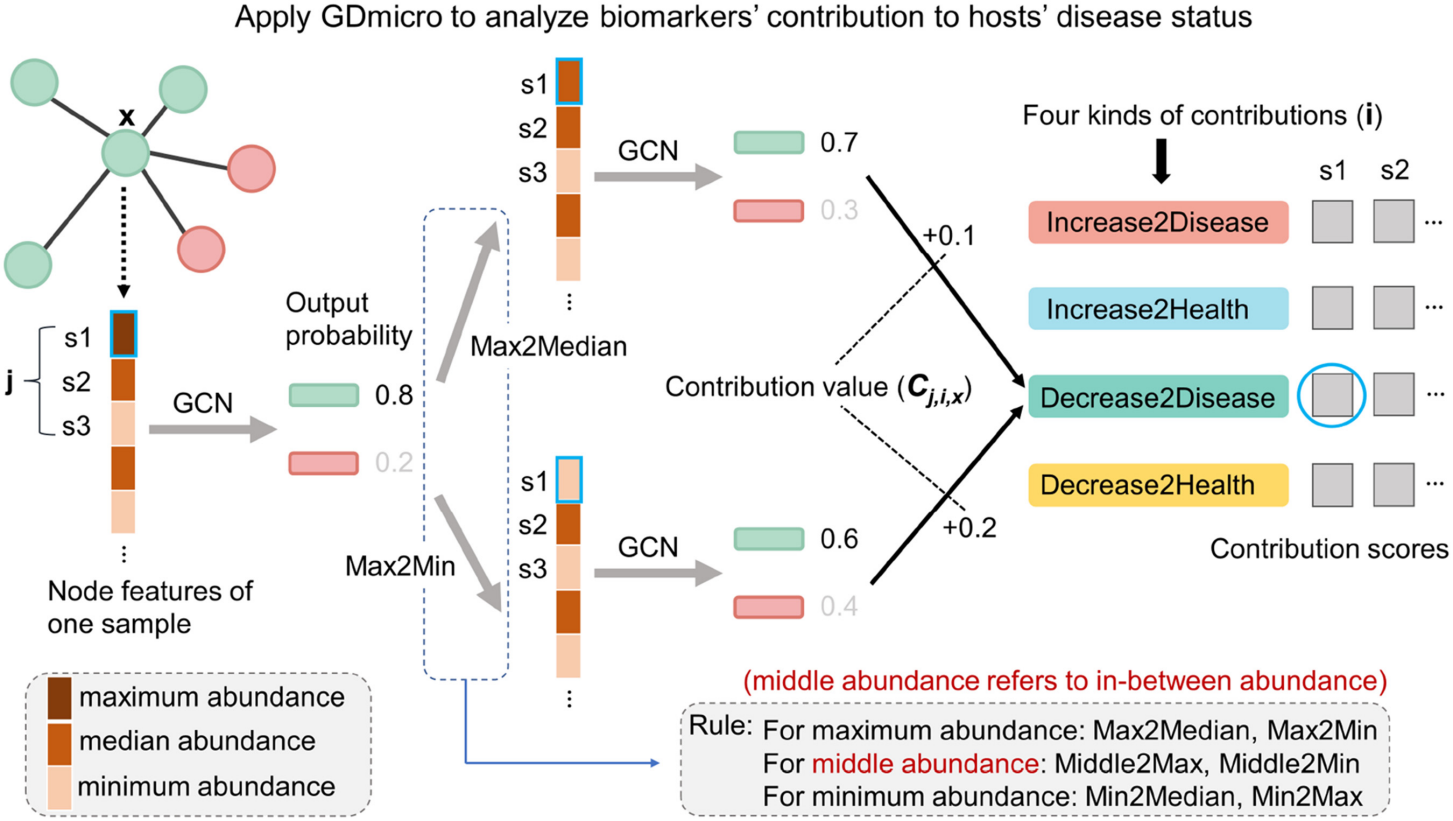
One example of using GDmicro to analyze a biomarker’s contribution to the host disease status. “s1, s2, s3:” identified biomarkers. “Rule:” the rule to change the raw abundance of the analyzed biomarker. For example, “Max2Median” means the raw abundance of the biomarker will change from the maximum value to the median value. “Contribution value:” the absolute value of the difference between the originally predicted probability of being healthy and the predicted probability of being healthy after changing the abundance.

Second, given the identified biomarkers, we will further characterize the contribution of these biomarkers to the hosts’ disease status. To solve this problem, we first define four kinds of contributions of biomarkers to the disease status: “Increase2Disease,” “Increase2Health,” “Decrease2Disease,” and “Decrease2Health.” There are four kinds of contributions because the microbes can have diverse effects on the disease status of the host. For instance, potential biomarkers usually exhibit a consistent change in abundance across patient samples in multiple studies, while other microbes demonstrate inconsistent alterations in abundance across different studies (Pittayanon *et al.* 2020). Consequently, by comparing the scores of these four contributions, users can comprehensively investigate the impact of specific microbes on the disease status of the host. These contributions reflect that modifying the abundance of certain species, either through increase or decrease, may result in a sample being more likely classified as diseased or healthy, respectively. For example, “Increase2Disease” represents that increasing the raw abundance of the species will result in a higher probability of the sample being classified as a diseased one. Thus, we can analyze a biomarker’s contribution by changing its raw abundance and checking the class (health or disease) probability predicted by the model. Based on the idea, we design a function utilizing the output probability of the GCN model to calculate the score for each kind of contribution. The higher the score, the more likely the biomarker has that specific contribution to the host’s disease status. Figure 2 illustrates the workflow of this function.

As shown in Fig. 2, the function initiates by recording the classification probability for each sample. Then, the program modifies the raw abundance of an identified biomarker in a sample and records the new classification probability under the modified abundance. Six modification rules are applied: “Max2Median,” “Max2Min,” “Middle2Max,” “Middle2Min,” “Min2Max,” and “Min2Median.” In these rules, “Middle” represents all in-between abundance in addition to the maximum and minimum abundance. Each rule represents the method of altering the raw abundance of the identified biomarker. For example, “Max2Median” indicates that the abundance of the selected biomarker is the maximum value among all species in the given sample, and will be adjusted from the maximum to the median value. With the original classification probabilities and those after applying various abundance modification rules, the function will compute the contribution value for each biomarker. The contribution value is calculated as the absolute difference between two probabilities: the probability of being healthy before and after modifying the abundance. This value reflects the biomarker’s contribution to disease status in the given sample. Lastly, the function calculates the contribution values of the identified biomarkers for all samples. Based on these values, the function determines the contribution score of one specific biomarker to the disease status of a host. The formula is $\sum_{x=1}^{n}\frac{C_{j,i,x}}{n}$, where *n* represents correctly predicted samples in the training data, *x* is the specific sample, *i* is the specific contribution type, *j* is the identified marker, and $C_{j,i,x}$ is the contribution value.

## 3 Results

In this section, we describe GDmicro’s results on 10-fold cross-validation and cross-study experiments in 10 cohorts with 5 diseases. In order to mimic real-world scenarios, we conducted these experiments using two different strategies for GDmicro. The first strategy, named “batch_run,” applies GDmicro to predict the host disease status for all the samples in the cohort altogether. In contrast, the second strategy, referred to as “single_run,” applies GDmicro to predict the host disease status for each individual separately, requiring multiple runs. Then, we analyzed the effect of different model architectures and parameters on the performance of GDmicro. Finally, we explored the ability of GDmicro to identify biomarkers for CRC and IBD cohorts and explore their influence on hosts’ disease status. We benchmarked four state-of-art tools, including SIAMCAT (Wirbel *et al.* 2021), MetAML (Pasolli *et al.* 2016), DeepMicro (Oh and Zhang 2020), and PopPhy-CNN (Reiman *et al.* 2020) with GDmicro in the 10-fold cross-validation and cross-study experiments. We take microbial compositional data as input for all of these tools. All tools are run with the recommended parameters or the best performance parameters reported in the corresponding paper.

### 3.1 Datasets and evaluation metrics

To benchmark with other tools (Pasolli *et al.* 2016, Oh and Zhang 2020, Rahman and Rangwala 2020, Reiman *et al.* 2020), we first used the same set of five cohorts covering five diseases: liver cirrhosis (Cirrhosis), CRC, IBD, obesity (Obesity), and type 2 diabetes (T2D). We then collected four CRC cohorts and one IBD cohort for the cross-study experiment. Finally, we used one additional CRC cohort, which is never used in any training or validation process, to evaluate the performance of GDmicro and other tools in real-world scenarios. These cohorts contain 1702 samples in total and have composition profiles available, which are generated by Metaphlan2 (Truong *et al.* 2015) or mOTU2 (Milanese *et al.* 2019). We downloaded five CRC cohorts from Wirbel *et al.* (2019) and all other cohorts from curatedMetagenomicData package (Pasolli *et al.* 2017). The detailed information of all cohorts is summarized in Table 1.

**Table 1.** The detailed information of 11 cohorts used in this work.^a^

Experiments	Cohort name	Disease	Country	Total samples	Unhealthy cases	Healthy controls	Ref.
10-fold cross-validation experiment	Cirrhosis	Liver cirrhosis	China	237	123	114	Qin *et al.* (2014)
	CRC-FR	Colorectal cancer	French	114	53	61	Zeller *et al.* (2014)
	IBD-DK	Inflammatory bowel disease	Denmark	110	25	85	Qin *et al.* (2010)
	Obesity	Obesity	Denmark	265	169	96	Le Chatelier *et al.* (2013)
	T2D	Type-2 diabetes	China	363	170	193	Qin *et al.* (2012)
Cross-study experiment	CRC-FR	Colorectal cancer	French	114	53	61	Zeller *et al.* (2014)
	CRC-DE	Colorectal cancer	Germany	120	60	60	Wirbel *et al.* (2019)
	CRC-CN	Colorectal cancer	China	128	74	54	Yu *et al.* (2017)
	CRC-US	Colorectal cancer	USA	104	52	52	Vogtmann *et al.* (2016)
	CRC-AT	Colorectal cancer	Austria	109	46	63	Feng *et al.* (2015)
	IBD-UK	Inflammatory bowel disease	UK	94	56	38	Ijaz *et al.* (2017)
	IBD-DK	Inflammatory bowel disease	Denmark	110	25	85	Qin *et al.* (2010)
Additional validation experiment	CRC-IN	Colorectal cancer	India	58	28	30	Gupta *et al.* (2019)

a The first five cohorts are used for the 10-fold cross-validation experiment. CRC-FR, IBD-DK, and the remaining five cohorts are used for the cross-study experiment. CRC-IN is used in the additional evaluation experiment.

Like many previous studies (Pasolli *et al.* 2016, Lo and Marculescu 2019, Nguyen and Zucker 2019, Zhu *et al.* 2019), we selected the AUC as the evaluation metric in this work, which summarizes the true positive and false positive rates and has a robust evaluation for the unequal ratio of each outcome. For the 10-fold cross-validation experiment, each experiment is repeated 10 times. Then, the margin of error for the mean of all experiments is calculated with a 95% confidence interval, and it is defined as:

(5) $$ME=t_{\left[1-\alpha /2,n-1\right]}\times \frac{\sigma_{s}}{\sqrt{n}},$$

where 1−α is the significance level, *n* is the sample size, $t_{\left[1-\alpha /2,n-1\right]}$ refers to critical value of *t*-distribution with degrees of freedom *n*−1 for an α/2 area of for the upper tail, and $\sigma_{s}$ is the sample standard deviation.

### 3.2 Ten-fold cross-validation experiment

To evaluate the performance of GDmicro, we applied all tools to classify disease status for samples from five cohorts (upper part in Table 1). We conducted 10-fold cross-validation using StratifiedKFold (*k* = 10) function in the sklearn package. The mean AUC and the margin or errors of all tools were recorded in Table 2. GDmicro with the “batch_run” strategy achieved the best performance in all tested cohorts. In particular, GDmicro-batch achieved 0.923 and 0.936 AUC in the CRC-FR and IBD-DK cohorts, which are 6.4% and 6.3% improvements over the second-ranked tool, respectively. In addition, while GDmicro-single does not match the performance of GDmicro-batch, it still outperforms the other tested methods. We further investigated the impact of the number of test samples on the performance, revealing that the model’s performance improves as the number of test samples increases (Supplementary Section S2.1 and Supplementary Fig. S1). Although some tools have competitive results, they have larger fluctuations than GDmicro. For example, while SIAMCAT achieved 0.949 AUC in the Cirrhosis cohort, its mean AUC in the T2D cohort was only 0.691. Similarly, DeepMicro had 0.940 AUC in the Cirrhosis cohort, but its mean AUC in the CRC-FR cohort was only 0.803, which was much lower than the top two tools. The results also reveal that the performance of all methods was not satisfactory in the Obesity and T2D cohorts. A possible reason is that the microbial features do not have strong correlations with the disease as reported in the previous study (LaPierre *et al.* 2019). Overall, GDmicro outperformed all other tools in the five tested cohorts.

**Table 2.** The mean AUC (10-fold cross-validation) of five tools on five popular disease cohorts.^a^

Cohorts	GDmicro-batch	GDmicro-single	SIAMCAT	MetAML	DeepMicro	PopPhy-CNN
Cirrhosis	**0.968 (0.0004)**	0.956 (0.001)	0.949 (0.0008)	0.945 (0.029)	0.94 (0.032)	0.94 (0.041)
CRC-FR	**0.923 (0.003)**	0.881 (0.005)	0.859 (0.003)	0.809 (0.053)	0.803 (0.077)	0.796 (0.075)
IBD-DK	**0.936 (0.004)**	0.882 (0.005)	0.867 (0.005)	0.873 (0.044)	0.863 (0.081)	0.781 (0.094)
Obesity	**0.741 (0.003)**	0.697 (0.003)	0.628 (0.005)	0.655 (0.071)	0.659 (0.068)	0.676 (0.074)
T2D	**0.816 (0.001)**	0.782 (0.001)	0.691 (0.002)	0.744 (0.048)	0.763 (0.064)	0.753 (0.055)

a The values in parenthesis refer to the margin of errors. Bold: the best performance of each cohort. Underlined: the performance of the second-ranked tool of each cohort. Notably, GDmicro-single has the second-best performance in the table, where underlining is used to highlight the best non-GDmicro approach. “GDmicro-batch:” run GDmicro once for all individuals in the validation sets. “GDmicro-single:” run GDmicro for one individual each time, requiring multiple runs.

### 3.3 Cross-study experiment

Test data in real-world applications usually come from different studies or domains from the training data. Thus, it is imperative to evaluate the robustness of the model across different domains. Because CRC and IBD are more associated with human gut microbes based on previous literature (Yu and Fang 2015, Rodríguez *et al.* 2020) and our experiments, we conducted cross-study experiments for these diseases.

**Figure 3.**
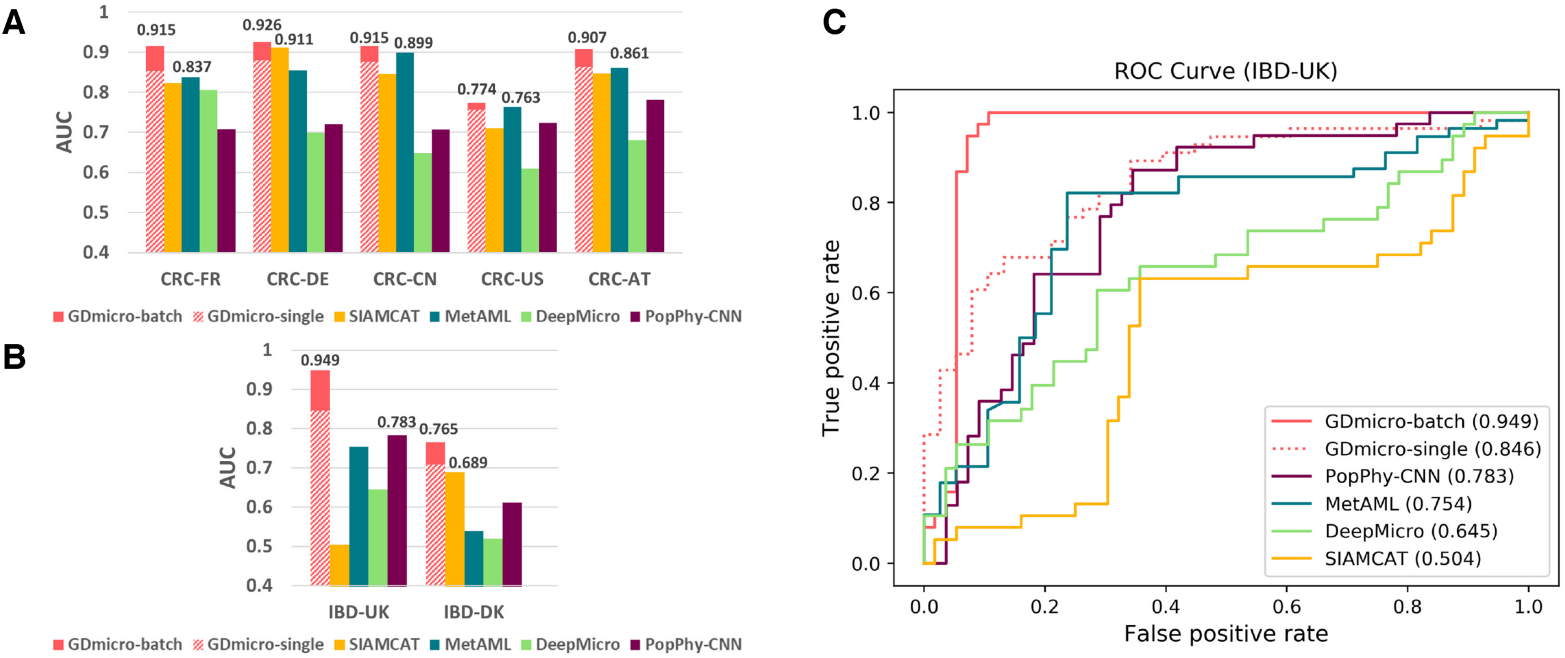
(A and B) The cross-study AUC of five tools in LOSO experiments of CRC (A) and IBD (B). The *X*-axis represents the test data in each LOSO experiment. For example, CRC-FR represents we train the model using CRC cohorts from four other countries and test on the CRC cohort from French. The values on the bar refer to the AUC of the best and second-best tools. “GDmicro-batch:” run GDmicro once for all individuals in the tested cohorts. “GDmicro-single:” run GDmicro for one individual each time, requiring multiple runs. The detailed AUC values of each tool are recorded in Supplementary Table S1. (C). ROC curves in the IBD-UK cohort. The value in the parentheses represents AUC for each tool.

We collected additional human gut microbiome data for CRC and IBD. The additional four CRC cohorts were all sampled from different individuals, while some samples in the additional IBD cohort were sampled from the same individual at different ages. In total, we have five CRC cohorts and two IBD cohorts. All these cohorts are from different countries. Then, we used the leave-one-study-out (LOSO) strategy to evaluate the performance of different tools. Specifically, we remove all samples from one study in the training data and retrain the model. Then, the model is tested on the removed samples. This setup mimics the real scenario where the query is from a different research group. The AUC of all tools in seven test cohorts was shown in Fig. 3.

Considering that both CRC-FR and IBD-DK cohorts were used in the 10-fold cross-validation experiment (Table 1), we first analyzed the performance change of different tools in these two cohorts. GDmicro, MetAML, and DeepMicro achieve comparable performance to the 10-fold cross-validation experiment in the CRC-FR cohort, while the AUC of SIAMCAT and PopPhy decreases more than 3%. However, the AUC of all tools decreases in the IBD-DK cohort. This result indicates that some models can still maintain good robustness in the cross-study experiments when sufficient training samples are available (e.g. 421 training samples for CRC-FR). However, when the training sample size is small, and some samples are from the same individual (e.g. 94 training samples from 50 individuals for IBD-DK), the classification of cross-study samples can be a challenge for all tested tools. Then, we will discuss the overall performance of these tested tools in all seven test cohorts.

As shown in Fig. 3A and B, GDmicro with the “batch_run” strategy achieved the best and most stable performance in the LOSO experiments. Compared to the second-best result, GDmicro-batch improved the AUC from 0.837 to 0.915 and 0.861 to 0.907 in CRC-FR and CRC-AT cohorts, respectively. MetAML and SIAMCAT achieved comparable performance in the CRC-US cohorts. However, their performance was not stable in the remaining cohorts. Although DeepMicro and PopPhy-CNN achieved satisfactory performance in the previous experiments, their performance was poor in most tested cohorts. In the two IBD cohorts, GDmicro-batch achieved more than 15% improvement in AUC in the IBD-UK cohort. Thus, we further draw a ROC curve of each tool in the IBD-UK cohort in Fig. 3C, which shows that GDmicro has more reliable classification results than other tools. However, all the tools do not perform well in the IBD-DK cohort, which contains data from the fewest number of individuals out of the seven cohorts. This result indicates that the performance of the learning-based methods may fluctuate with the change of the training cohort size. Nevertheless, GDmicro achieves more than 5% improvement in AUC.

In line with the results from the 10-fold cross-validation experiment, GDmicro with the “single_run” strategy has decreased performance compared to GDmicro-batch. When there are multiple samples, the edges between them or the increased number of edges between training and test samples can help the semi-supervised learning of GCN, which might explain the performance difference between the two scenarios. Despite the lower AUC, GDmicro-single outperforms all other tools on the two IBD cohorts. In addition, its average AUC achieved a 4% improvement compared to the second-best tool across all tested cohorts (Supplementary Table S1). Similar to the previous experiment, we also investigated the influence of test sample size on the cross-study experiment (Supplementary Section S2.1). The result shows that GDmicro exhibited improved performance with an increase in the number of test samples (Supplementary Fig. S2). Especially, the performance has a rapid convergence as the number of test samples increases in some tested cohorts.

From Fig. 3A and B, we also observed that machine-learning-based tools (MetAML and SIAMCAT) tend to have better performance than deep-learning-based tools (DeepMicro and PopPhy-CNN) in most LOSO experiments. This result indicated that deep-learning-based tools are more likely to overfit the training set because of the complexity of the model. However, by using a deep adaptation network to learn the transferable latent features, GDmicro maintains good generalization capabilities in the cross-study host disease status classification task.

### 3.4 Ablation study and parameter analysis

In this experiment, we study how different architectures and parameters influence the performance of GDmicro using ablation study and parameter analysis. Specifically, we analyzed the influence of the adaptation loss function, GCN model, and hyper-parameter *k* in the *k*NN graph on the performance of GDmicro. To be more consistent with the usage of real-world data, we analyzed datasets of the cross-study experiment. The analysis results show that the current architecture outperforms all other tested architectures, demonstrating the efficiency of domain adaptation and the GCN model (Supplementary Fig. S3A). In addition, we also found that the performance of GDmicro is not very sensitive to *k* (Supplementary Fig. S3B). By default, we use k=5 to construct the *k*NN graph. Additional details regarding this experiment can be found in Supplementary Section S2.2.

### 3.5 GDmicro identifies important biomarkers and analyzes their contribution to CRC and IBD

In this experiment, we applied GDmicro to identify important biomarkers and explore their contribution to hosts’ disease status from cohorts used in the cross-study experiment. It should be noted that the goal of this experiment is to identify potential biomarkers, and it is a common practice to conduct biomarker discovery using a batch of samples (Segata *et al.* 2011, Liu *et al.* 2022). Thus, we run GDmicro with the “batch_run” mode in this experiment. As mentioned in Section 2, GDmicro could identify important biomarkers using a performance-based method. According to the method, the species with better classification ability will have higher AUC. Therefore, we can evaluate the classification ability of all species in the given data and record their AUC. Then, we selected the top 10 species with the highest AUC values for further analysis. The identified biomarkers and their relative abundance distribution are shown in Supplementary Fig. S4. We observed that many identified species of CRC cohorts were enriched in CRC samples, and their relative abundance was also higher. Unlike CRC, many identified species in the IBD cohort were enriched in healthy samples, which was in line with previous observations (Ma *et al.* 2021, Olbjørn *et al.* 2022). Given that abundance-based statistical analysis is a prevalent method for discovering microbial biomarkers, we examined the differences between biomarkers identified by GDmicro and those detected using the Wilcoxon test, a popular statistics-based approach. The details about using the Wilcoxon test to discover biomarkers are described in Supplementary Section S2.3. In the following paragraphs, we first analyzed the biomarkers identified by GDmicro and the Wilcoxon test in terms of robustness, discriminatory capabilities, and cross-study consistency. Then, we investigated the contribution of these cross-study biomarkers to the host disease status.

**Figure 4.**
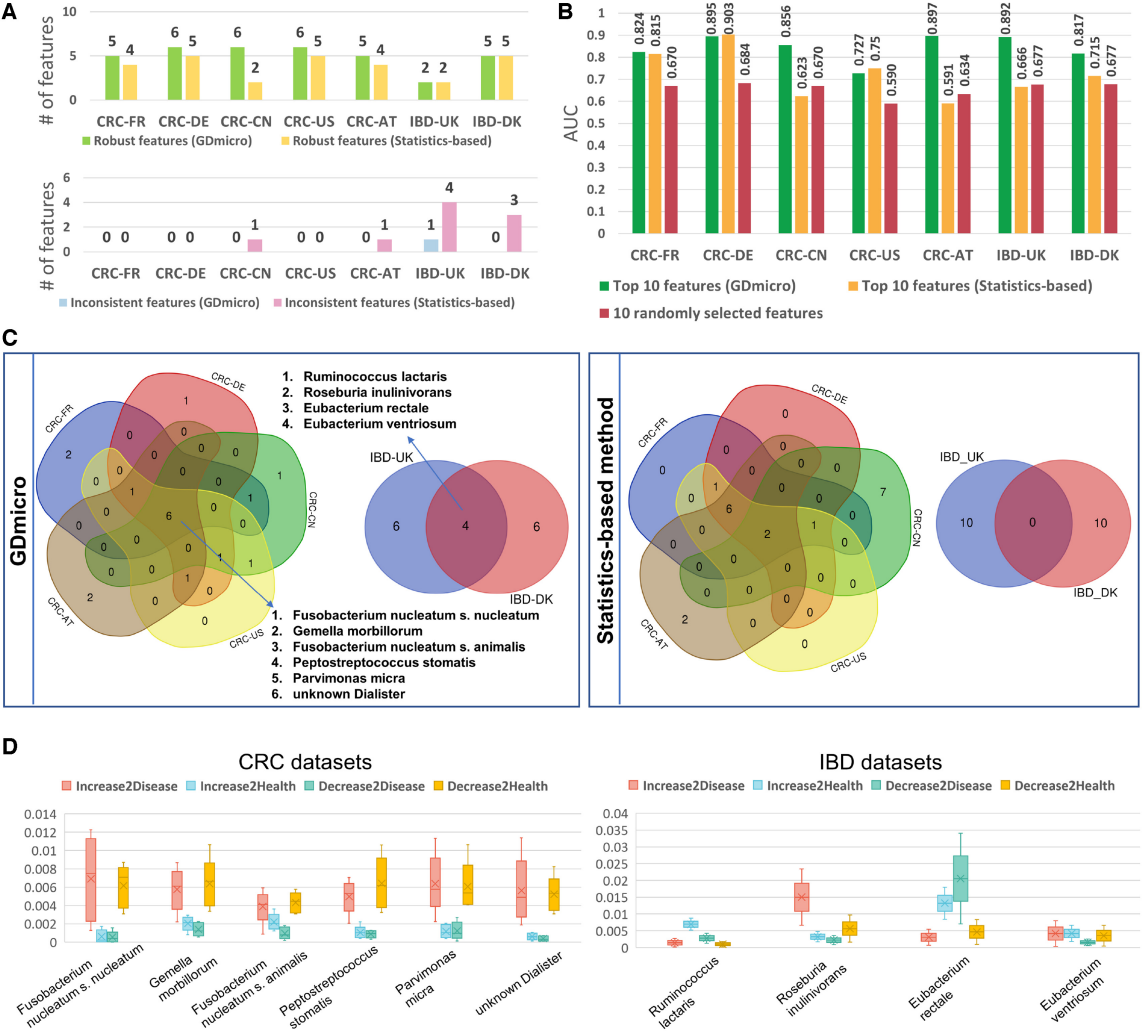
(A). The number of robust and inconsistent features identified by GDmicro and the statistics-based method (Wilcoxon test). (B). AUC comparison across studies in the LOSO experiment using the top 10 features identified by three different methods. The AUC of “10 randomly selected features” is the average AUC of five-time repeated experiments. (C). Venn diagrams for the identified top 10 biomarkers in different cohorts by GDmicro and the statistics-based method. (D). The boxplot of contribution scores of cross-study biomarkers in CRC and IBD datasets. The *Y*-axis represents the contribution score values of the biomarker in five CRC and two IBD cohorts.

To assess the robustness of identified biomarkers, we further calculated the *P*-value of identified features in each study (Supplementary Fig. S5) and defined two kinds of features. The first feature is called “robust feature” that exhibits a consistent positive or negative *P*-value of <0.05 across all studies. The second type of feature, called “inconsistent feature,” refers to features that exhibit both positive and negative *P*-values across different datasets. In real-world applications, we expect that effective biomarker discovery methods can uncover more robust features while reducing the number of inconsistent features. As Fig. 4A shows, GDmicro identified more robust features and produced fewer inconsistent features than the statistics-based method.

To evaluate the discriminatory capabilities of the identified biomarkers, we conducted the LOSO experiment using only the top 10 species selected by GDmicro and the Wilcoxon test, respectively. For comparison, we randomly selected 10 features from each study and reran the LOSO experiment, repeating this five times to avoid data bias. As shown in Fig. 4B, GDmicro consistently achieved higher AUC values in most tested datasets than the Wilcoxon test and outperformed the set with randomly selected species in all datasets. This result shows that the top-ranked species identified by GDmicro have better discriminatory capabilities, and an additional experiment further confirmed this superiority when using the top 50 selected features (Supplementary Section S2.3 and Supplementary Fig. S6).

Previous studies have reported that biomarkers with cross-study consistency (aka cross-study biomarkers) are crucial for disease identification and are valuable for clinical diagnosis (Wirbel *et al.* 2019, Zhang *et al.* 2022). Thus, we compared the top 10 biomarkers identified by GDmicro and the Wilcoxon test across studies. Figure 4C shows that GDmicro identified six cross-study biomarkers for CRC and four for IBD, while the Wilcoxon test detected only two for CRC. Many cross-study biomarkers identified by GDmicro are highly associated with CRC and IBD (Zeller *et al.* 2014, Zhang *et al.* 2017, Kwong *et al.* 2018). For example, *Gemella morbillorum* and *Parvimonas micra* are reported as biomarkers for non-invasive diagnosis of CRC (Yao *et al.* 2021), while *Eubacterium rectale*, a beneficial bacteria, decreases in IBD patients (Qiu *et al.* 2022). To know how these cross-study biomarkers affect hosts’ disease status, we plotted their contribution scores, as calculated by GDmicro, in Fig. 4D. The result shows that all CRC biomarkers may promote CRC, consistent with previous observations (Wirbel *et al.* 2019). Conversely, IBD biomarkers play different roles: *Ruminococcus lactaris* and *E.rectale* tend to inhibit IBD, while the remaining two species prompt it. These results also align with the four species’ relative abundance distribution (Supplementary Fig. S4).

### 3.6 Evaluate GDmicro on one additional CRC dataset

**Figure 5.**
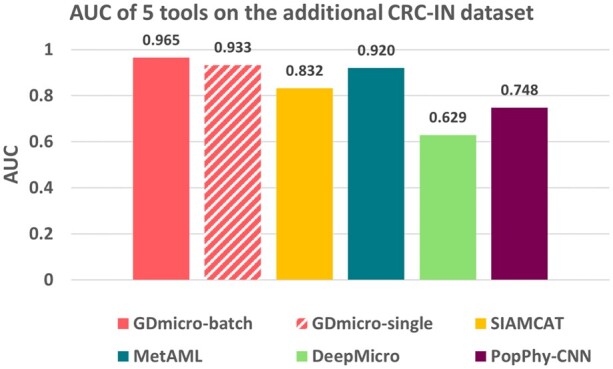
The AUC of five tools on the additional CRC dataset. The model is trained on five CRC datasets used in the cross-study experiment. “GDmicro-batch:” run GDmicro once for all individuals in the cohort. “GDmicro-single:” run GDmicro for one individual each time, requiring 58 runs.

To further assess the robustness of GDmicro and its applicability in real-world scenarios, we conducted an additional evaluation using a distinct CRC dataset named “CRC-IN” (Gupta *et al.* 2019), which consisted of 58 samples not utilized in previous training or validation processes. In this experiment, the model was trained on the five CRC datasets used in the cross-study experiment, then it was employed to predict the labels of samples from the additional dataset. To mimic the real-world scenarios, we still use two different strategies for GDmicro in this experiment. As shown in Fig. 5, GDmicro with different running strategies outperforms other tools. While MetAML demonstrated competitive performance, its effectiveness on the IBD datasets in the cross-study experiment was unsatisfactory. In addition, consistent with 10-fold cross-validation and cross-study experiments, GDmicro with the “batch_run” strategy outperformed the “single_run” strategy. However, it is important to note that GDmicro still outperforms all other tested tools in both scenarios. Overall, this experiment further reinforces the superior robustness of GDmicro compared to other tools.

## 4 Discussion

In this work, we demonstrate that GDmicro outperforms the state-of-art tools in classifying host disease status. To reduce the domain discrepancy between data, we use a deep adaptation network to learn the transferable latent features from compositional abundance data. Then, we build an inter-host microbiome similarity graph and apply a GCN model to integrate structural and abundance features and utilize information from unlabeled and limited labeled samples. As a result, GDmicro can achieve more accurate and robust host disease status classification. Furthermore, GDmicro demonstrates superior accuracy in identifying disease-related species compared to the statistics-based method, and it elucidates their contribution to the host’s disease status. This insight offers valuable information for biomarker discovery.

Although GDmicro has improved host disease status classification, it has three major limitations. First, when the graph contains few test samples or only a single test sample, the GCN model cannot fully leverage its advantage to effectively utilize the features from the test samples. Consequently, this limitation can lead to a potential decrease in performance. Second, GDmicro is designed to classify a single disease at a time. If a sample exhibits multiple diseases, GDmicro is unable to simultaneously identify all of these diseases in a single classification. Finally, it is worth mentioning that GDmicro does not possess a specific function optimized for the disease classification of time-series samples, which is a highly relevant task within this field of research.

Thus, we have two goals to optimize in future work. First, current efforts are aimed at classifying whether a host has a particular disease or not. However, in practical applications, input samples may suffer from multiple diseases. Therefore, we intend to combine the graphs of different diseases for GCN to explore whether GDmicro can classify multiple diseases at the same time. Second, we will consider extending GDmicro to accommodate temporal longitudinal microbiome data. To support such an extension, methods like Long Short-Term Memory Networks will be integrated into our current architecture for feature extraction and classification.

## Supplementary Material

btad747_Supplementary_Data

## Data Availability

The source code of GDmicro and all datasets used in this work are freely available at https://github.com/liaoherui/GDmicro.

## References

[btad747-B1] Abdill RJ , Adamowicz EM, Blekhman R et al Public human microbiome data are dominated by highly developed countries. PLoS Biol 2022;20:e3001536. 35167588 10.1371/journal.pbio.3001536 PMC8846514

[btad747-B2] Chen J , Chia N, Kalari KR et al Multiple sclerosis patients have a distinct gut microbiota compared to healthy controls. Sci Rep 2016;6:28484. 27346372 10.1038/srep28484 PMC4921909

[btad747-B3] Curry KD , Nute MG, Treangen TJ et al It takes guts to learn: machine learning techniques for disease detection from the gut microbiome. Emerg Top Life Sci 2021;5:815–27. 34779841 10.1042/ETLS20210213 PMC8786294

[btad747-B4] Feng Q , Liang S, Jia H et al Gut microbiome development along the colorectal adenoma-carcinoma sequence. Nat Commun 2015;6:6528. 25758642 10.1038/ncomms7528

[btad747-B5] Gao Z , Shi J, Wang J. GQ-GCN: group quadratic graph convolutional network for classification of histopathological images. In: *Medical Image Computing and Computer Assisted Intervention – MICCAI 2021*, *Strasbourg, France*. Berlin, Heidelberg: Springer-Verlag. 121–31. 2021.

[btad747-B6] Gligorijević V , Renfrew PD, Kosciolek T et al Structure-based protein function prediction using graph convolutional networks. Nat Commun 2021;12:3168. 34039967 10.1038/s41467-021-23303-9 PMC8155034

[btad747-B7] Gomaa EZ. Human gut microbiota/microbiome in health and diseases: a review. Antonie Van Leeuwenhoek 2020;113:2019–40. 33136284 10.1007/s10482-020-01474-7

[btad747-B8] Gretton A , Sejdinovic D, Strathmann H et alOptimal kernel choice for large-scale two-sample tests. In: Pereira F, Burges CJ, Bottou L et al (eds), Advances in Neural Information Processing Systems, *Lake Tahoe, Nevada, USA*. Vol. 25. Curran Associates, Inc., 1214–22. 2012.

[btad747-B9] Gupta A , Dhakan DB, Maji A et al Association of *Flavonifractor plautii*, a flavonoid-degrading bacterium, with the gut microbiome of colorectal cancer patients in India. mSystems 2019;4:e00438-19. 31719139 10.1128/mSystems.00438-19 PMC7407896

[btad747-B10] Han P , Yang P, Zhao P et al GCN-MF: disease-gene association identification by graph convolutional networks and matrix factorization. In: *Proc. ACM SIGKDD Conference on Knowledge Discovery and Data Mining, Anchorage AK USA*. New York, United States: Association for Computing Machinery. ISBN 9781450362016. 705–13. 2019.

[btad747-B11] He Y , Wu W, Zheng H-M et al Regional variation limits applications of healthy gut microbiome reference ranges and disease models. Nat Med 2018;24:1532–5. 30150716 10.1038/s41591-018-0164-x

[btad747-B12] Ijaz UZ , Quince C, Hanske L et al The distinct features of microbial ‘dysbiosis’ of Crohn’s disease do not occur to the same extent in their unaffected, genetically-linked kindred. PLoS One 2017;12:e0172605. 28222161 10.1371/journal.pone.0172605 PMC5319678

[btad747-B13] Kipf TN , Welling M. Semi-supervised classification with graph convolutional networks. In: *International Conference on Learning Representations*, *Toulon, France*. New York, United States: Curran Associates-Red Hook. 2017.

[btad747-B14] Kwong TNY , Wang X, Nakatsu G et al Association between bacteremia from specific microbes and subsequent diagnosis of colorectal cancer. Gastroenterology 2018;155:383–90.e8. 29729257 10.1053/j.gastro.2018.04.028

[btad747-B15] LaPierre N , Ju CJ-T, Zhou G et al MetaPheno: a critical evaluation of deep learning and machine learning in metagenome-based disease prediction. Methods 2019;166:74–82. 30885720 10.1016/j.ymeth.2019.03.003 PMC6708502

[btad747-B16] Le Chatelier E , Nielsen T, Qin J et al; MetaHIT consortium. Richness of human gut microbiome correlates with metabolic markers. Nature 2013;500:541–6. 23985870 10.1038/nature12506

[btad747-B17] Li X , Ma J, Leng L et al MoGCN: a multi-omics integration method based on graph convolutional network for cancer subtype analysis. Front Genet 2022;13:806842. 35186034 10.3389/fgene.2022.806842 PMC8847688

[btad747-B18] Liu Y , Zhu J, Wang H et al Machine learning framework for gut microbiome biomarkers discovery and modulation analysis in large-scale obese population. BMC Genomics 2022;23:850. 36564713 10.1186/s12864-022-09087-2 PMC9789565

[btad747-B19] Lloyd-Price J , Arze C, Ananthakrishnan AN et al; IBDMDB Investigators. Multi-omics of the gut microbial ecosystem in inflammatory bowel diseases. Nature 2019;569:655–62. 31142855 10.1038/s41586-019-1237-9 PMC6650278

[btad747-B20] Lo C , Marculescu R. MetaNN: accurate classification of host phenotypes from metagenomic data using neural networks. BMC Bioinformatics 2019;20:314. 31216991 10.1186/s12859-019-2833-2 PMC6584521

[btad747-B21] Long M , Cao Y, Wang J et al Learning transferable features with deep adaptation networks. In: *Proceedings of the 32nd International Conference on International Conference on Machine Learning, Lille France, Vol. 37*. Massachusetts, United States: JMLR-Norfolk. 97–105. 2015.

[btad747-B22] Ma Y , Zhang Y, Xiang J et al Metagenome analysis of intestinal bacteria in healthy people, patients with inflammatory bowel disease and colorectal cancer. Front Cell Infect Microbiol 2021;11:599734. 33738265 10.3389/fcimb.2021.599734 PMC7962608

[btad747-B23] Manichanh C , Rigottier-Gois L, Bonnaud E et al Reduced diversity of faecal microbiota in Crohn’s disease revealed by a metagenomic approach. Gut 2006;55:205–11. 16188921 10.1136/gut.2005.073817 PMC1856500

[btad747-B24] Milanese A , Mende DR, Paoli L et al Microbial abundance, activity and population genomic profiling with mOTUs2. Nat Commun 2019;10:1014. 30833550 10.1038/s41467-019-08844-4 PMC6399450

[btad747-B25] Nguyen TH , Zucker J-D. Enhancing metagenome-based disease prediction by unsupervised binning approaches. In: *2019 11th International Conference on Knowledge and Systems Engineering (KSE)*, *Da Nang, Vietnam*. New York City, United States: IEEE. 1–5. 2019.

[btad747-B26] Oh M , Zhang L. DeepMicro: deep representation learning for disease prediction based on microbiome data. Sci Rep 2020;10:6026. 32265477 10.1038/s41598-020-63159-5 PMC7138789

[btad747-B27] Olbjørn C , Småstuen MC, Moen AEF et al Targeted analysis of the gut microbiome for diagnosis, prognosis and treatment individualization in pediatric inflammatory bowel disease. Microorganisms 2022;10:1273. 35888992 10.3390/microorganisms10071273 PMC9319120

[btad747-B28] Palmas V , Pisanu S, Madau V et al Gut microbiota markers associated with obesity and overweight in Italian adults. Sci Rep 2021;11:5532. 33750881 10.1038/s41598-021-84928-w PMC7943584

[btad747-B29] Pasolli E , Schiffer L, Manghi P et al Accessible, curated metagenomic data through ExperimentHub. Nat Methods 2017;14:1023–4. 29088129 10.1038/nmeth.4468 PMC5862039

[btad747-B30] Pasolli E , Truong DT, Malik F et al Machine learning meta-analysis of large metagenomic datasets: tools and biological insights. PLoS Comput Biol 2016;12:e1004977. 27400279 10.1371/journal.pcbi.1004977 PMC4939962

[btad747-B31] Pittayanon R , Lau JT, Leontiadis GI et al Differences in gut microbiota in patients with vs without inflammatory bowel diseases: a systematic review. Gastroenterology 2020;158:930–46.e1. 31812509 10.1053/j.gastro.2019.11.294

[btad747-B32] Qin J , Li R, Raes J et al; MetaHIT Consortium. A human gut microbial gene catalogue established by metagenomic sequencing. Nature 2010;464:59–65. 20203603 10.1038/nature08821 PMC3779803

[btad747-B33] Qin J , Li Y, Cai Z et al A metagenome-wide association study of gut microbiota in type 2 diabetes. Nature 2012;490:55–60. 23023125 10.1038/nature11450

[btad747-B34] Qin N , Yang F, Li A et al Alterations of the human gut microbiome in liver cirrhosis. Nature 2014;513:59–64. 25079328 10.1038/nature13568

[btad747-B35] Qiu P , Ishimoto T, Fu L et al The gut microbiota in inflammatory bowel disease. Front Cell Infect Microbiol 2022;12:733992. 35273921 10.3389/fcimb.2022.733992 PMC8902753

[btad747-B36] Rahman MA , Rangwala H. IDMIL: an alignment-free interpretable deep multiple instance learning (MIL) for predicting disease from whole-metagenomic data. Bioinformatics 2020;36:i39–47. 32657370 10.1093/bioinformatics/btaa477 PMC7355246

[btad747-B37] Reiman D , Metwally AA, Sun J et al PopPhy-CNN: a phylogenetic tree embedded architecture for convolutional neural networks to predict host phenotype from metagenomic data. IEEE J Biomed Health Inform 2020;24:2993–3001. 32396115 10.1109/JBHI.2020.2993761

[btad747-B38] Rodríguez C , Romero E, Garrido-Sanchez L et al Microbiota insights in CLOSTRIDIUM DIFFICILE infection and inflammatory bowel disease. Gut Microbes 2020;12:1725220. 32129694 10.1080/19490976.2020.1725220 PMC7524151

[btad747-B39] Segata N , Izard J, Waldron L et al Metagenomic biomarker discovery and explanation. Genome Biol 2011;12:R60. 21702898 10.1186/gb-2011-12-6-r60 PMC3218848

[btad747-B40] Shang J , Jiang J, Sun Y et al Bacteriophage classification for assembled contigs using graph convolutional network. Bioinformatics 2021;37:i25–33. 34252923 10.1093/bioinformatics/btab293 PMC8275337

[btad747-B41] Shen Y , Zhu J, Deng Z et al EnsDeepDP: an ensemble deep learning approach for disease prediction through metagenomics. IEEE/ACM Trans Comput Biol Bioinform 2022;20:986–98. 10.1109/TCBB.2022.3201295 36001521

[btad747-B42] Truong DT , Franzosa EA, Tickle TL et al MetaPhlAn2 for enhanced metagenomic taxonomic profiling. Nat Methods 2015;12:902–3. 26418763 10.1038/nmeth.3589

[btad747-B43] van Engelen JE , Hoos HH. A survey on semi-supervised learning. Mach Learn 2020;109:373–440.

[btad747-B44] Vogtmann E , Hua X, Zeller G et al Colorectal cancer and the human gut microbiome: reproducibility with whole-genome shotgun sequencing. PLoS One 2016;11:e0155362. 27171425 10.1371/journal.pone.0155362 PMC4865240

[btad747-B45] Wang T , Shao W, Huang Z et al MOGONET integrates multi-omics data using graph convolutional networks allowing patient classification and biomarker identification. Nat Commun 2021;12:3445. 34103512 10.1038/s41467-021-23774-w PMC8187432

[btad747-B46] Wirbel J , Pyl PT, Kartal E et al Meta-analysis of fecal metagenomes reveals global microbial signatures that are specific for colorectal cancer. Nat Med 2019;25:679–89. 30936547 10.1038/s41591-019-0406-6 PMC7984229

[btad747-B47] Wirbel J , Zych K, Essex M et al Microbiome meta-analysis and cross-disease comparison enabled by the SIAMCAT machine learning toolbox. Genome Biol 2021;22:93. 33785070 10.1186/s13059-021-02306-1 PMC8008609

[btad747-B48] Yao L , Mao C, Luo Y. Graph convolutional networks for text classification. AAAI 2019;33:7370–7.

[btad747-B49] Yao Y , Ni H, Wang X et al A new biomarker of fecal bacteria for non-invasive diagnosis of colorectal cancer. Front Cell Infect Microbiol 2021;11:744049. 34976850 10.3389/fcimb.2021.744049 PMC8719628

[btad747-B50] Yu J , Feng Q, Wong SH et al Metagenomic analysis of faecal microbiome as a tool towards targeted non-invasive biomarkers for colorectal cancer. Gut 2017;66:70–8. 26408641 10.1136/gutjnl-2015-309800

[btad747-B51] Yu YN , Fang JY. Gut microbiota and colorectal cancer. Gastrointest Tumors 2015;2:26–32. 26674881 10.1159/000380892 PMC4668798

[btad747-B52] Yu Z , Huang F, Zhang X et al. Predicting drug-disease associations through layer attention graph convolutional network. Brief Bioinform 2021;22:bbaa243. 33078832 10.1093/bib/bbaa243

[btad747-B53] Zeller G , Tap J, Voigt A et al Potential of fecal microbiota for early-stage detection of colorectal cancer. Mol Syst Biol 2014;10:766. 25432777 10.15252/msb.20145645 PMC4299606

[btad747-B54] Zhang H , Wu J, Ji D et al Microbiome analysis reveals universal diagnostic biomarkers for colorectal cancer across populations and technologies. Front Microbiol 2022;13:1005201. 36406447 10.3389/fmicb.2022.1005201 PMC9668862

[btad747-B55] Zhang S-L , Wang S-N, Miao C-Y et al Influence of microbiota on intestinal immune system in ulcerative colitis and its intervention. Front Immunol 2017;8:1674. 29234327 10.3389/fimmu.2017.01674 PMC5712343

[btad747-B56] Zhou J , Cui G, Hu S et al Graph neural networks: a review of methods and applications. AI Open 2020;1:57–81.

[btad747-B57] Zhu X , Ghahramani Z, Lafferty J. Semi-supervised learning using Gaussian fields and harmonic functions. In: *Proceedings of the Twentieth International Conference on Machine Learning*, *Washington, DC, USA*. 912–9. Washington DC, USA: AAAI Press, 2003.

[btad747-B58] Zhu Z , Ren J, Michail S et al MicroPro: using metagenomic unmapped reads to provide insights into human microbiota and disease associations. Genome Biol 2019;20:154. 31387630 10.1186/s13059-019-1773-5 PMC6683435

