# Supplementary material for: GDmicro: classifying host disease status with GCN and deep adaptation network based on the human gut microbiome data
Source: Bioinformatics. 2023 Dec 12;39(12):btad747. doi: 10.1093/bioinformatics/btad747 (PMC10749762; doi:10.1093/bioinformatics/btad747)
Supplement: btad747_Supplementary_Data [file btad747_supplementary_data.pdf]

Supplementary information for "GDmicro: classifying host disease status with GCN and Deep adaptation network based on the human gut microbiome data"

Herui Liao, Jiayu Shang, and Yanni Sun

Department of Electrical Engineering, City University of Hong Kong, Kowloon, Hong Kong SAR

December 6, 2023

1 Supplementary Methods

1.1 MK-MMD-based adaptation regularizer

In equation (1) of the main article, d_k^2 is the squared formulation of MK-MMD. Suppose the source domain and target domain are characterized by probability distributions p and q, then the d_k^2 is defined as:

$$d_k^2(D_s, D_t) \triangleq \|E_p\left[\phi\left(x^s\right)\right] - E_q[\phi\left(x^t\right)]\|_{\mathcal{H}_k}^2, \quad (1)$$

$$\text{where } E_{x \sim p} f(x) = \langle f(x), \mu_k(p) \rangle_{\mathcal{H}_k}, \quad (2)$$

where ϕ is hidden layers of the network, $\mathcal{H}_k$ is the reproducing kernel Hilbert space endowed with a characteristic kernel k, and $\mu_k(p)$ is the mean embedding of distribution p in $\mathcal{H}_k$. The main purpose of MK-MMD is to minimize the d_k^2 such that the two domain distribution p and q become closer. When $d_k^2 = 0$, p will be equal to q. Thus, the MK-MMD is able to reflect the discrepancy between the source and target. As a result, by minimizing the loss function with the MK-MMD-based adaptation regularizer, the model can learn the transferable latent features between data from different domains.

2 Supplementary Experiments

2.1 Analyzing the influence of test sample size on GDmicro's performance

In this experiment, we investigated the influence of test sample size on the model's performance. To achieve this, we run GDmicro on test sets of different sizes, including 1 (named as "single"), 3, 5, half of all samples (named as "half"), and all samples (named as "batch"). For test sample size n between single and batch, considering all combinations can lead to tedious setup and long running time. Thus, we randomly selected n samples from all and repeated this process 50 times. Then, we report the average AUC for these selections.

We first explored the influence of test sample size on the 10-fold cross-validation experiment. As shown in Supplementary Figure S1, GDmicro's performance consistently improves as the number of input test samples increases across all tested cohorts. Especially, the improvement was significant when the number of test samples increased from 5 to half and from half to all for most tested cohorts. However, the performance on the CRC-FR and IBD-DK cohorts does not exhibit significant improvement when we increase the number of test samples from 5 to half, in contrast to the improvement observed when increasing from half to all. This discrepancy could be attributed to the limited total number of test samples, resulting in a similar test sample size between the 5 and half groups for these two cohorts.

Then, we further investigated the influence of test sample size on the cross-study experiment. As shown in Supplementary Figure S2, GDmicro still has improved performance with an increase in the number of test samples. On

CRC-DE and CRC-AT cohorts, we also notice that the performance has a rapid convergence when the number of test samples increases to three. In contrast, the performance of the remaining cohorts displays varying changes but exhibits an overall improvement. These experiments show the significance of test sample size in enhancing the performance of GDmicro and highlight the advantages of increasing cohort size for achieving better results.

2.2 Ablation study and parameter analysis

In this experiment, we study how different architectures and parameters influence the performance of GDmicro using ablation study and parameter analysis. Specifically, we analyzed the influence of the adaptation loss function, GCN model, and hyper-parameter k in the kNN graph on the performance of GDmicro. To be more consistent with the usage of real-world data, we analyzed datasets of the cross-study experiment.

As discussed in the Methods section, the loss function used in the deep adaptation network is based on multiple kernel variants of maximum mean discrepancies (MK-MMD), which aims to reduce domain discrepancy between data from different studies. To show the effect of different loss functions on the performance of GDmicro, we repeated the analysis in the cross-study experiment with and without MK-MMD-based loss. When the loss function only contains the cross-entropy loss, the model is a multi-layer fully connected network (aka multi-layer perceptron or MLP) that ignores the domain discrepancy. In addition, to know whether the GCN model improved the classification performance, we also combined the deep adaptation network and MLP for host disease status classification in the cross-study experiment.

As shown in Supplementary Figure S3A, GDmicro with default architecture achieved better performance than the model without domain adaptation in five out of seven tested datasets. This result indicates that the MK-MMD-based loss improves the model's robustness by learning transferable latent features. Related to this, the deep adaptation network outperformed MLP in all tested cohorts, which demonstrated the deep adaptation network improved the classification robustness by minimizing the domain discrepancy. We also noticed that GDmicro with default architecture achieved better performance than the single deep adaptation network and MLP, demonstrating that the GCN model improved the classification AUC by incorporating structural and compositional abundance features and utilizing information from unlabeled samples.

The hyper-parameter k is an important parameter for the kNN graph, which determines the graph's topological structure. Thus, we investigated the performance of GDmicro under different k by repeating the analysis in the cross-study experiment with $k \in \{3, 5, 7, 10\}$. Supplementary Figure S3B shows the performance of GDmicro when k varies from 3 to 10. As shown in Supplementary Figure S3B, the performance of GDmicro doesn't fluctuate much in all tested datasets with the change of k, which indicates that GDmicro is not very sensitive to k. By default, we use $k = 5$ to construct the kNN graph.

2.3 LOSO experiments with top 50 features selected by different methods

To identify biomarkers with the Wilcoxon test, we calculated the p-value of each feature using all the training data. In this experiment, a positive p-value signifies that the feature is enriched in disease samples, whereas a negative value indicates enrichment in healthy samples. Subsequently, all features are sorted from smallest to largest based on the absolute value of their p-values. To avoid data bias, we repeated the LOSO experiment with the top 50 features identified by GDmicro and the statistics-based method. The result shows that the average AUC for GDmicro is 0.891, while the average AUC for the statistics-based method is 0.869 (Supplementary Figure S6), a finding consistent with the result observed for the top 10 features.

3 Supplementary Figures

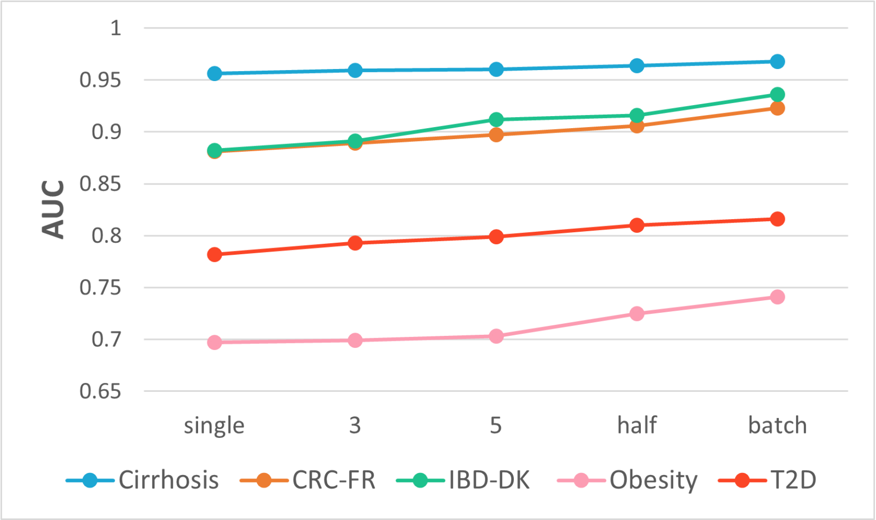

Supplementary Figure S1. The AUC of GDmicro across five disease cohorts under varying numbers of input test samples. "Single": run GDmicro on each individual sample separately. "Half": run GDmicro on half of the samples in one experiment. "Batch": run GDmicro on all samples altogether.

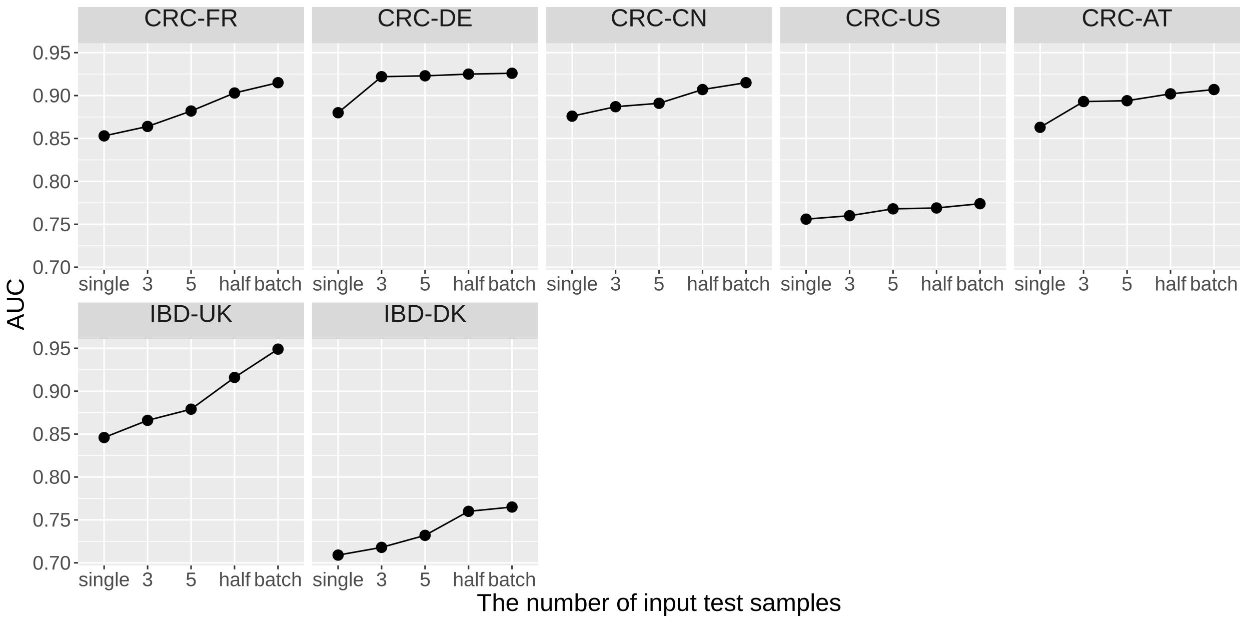

Supplementary Figure S2. The AUC of GDmicro across different numbers of input test samples in the Leave-One-Study-Out (LOSO) experiment. "Single": run GDmicro on each individual sample separately. "Half": run GDmicro on half of the samples in one experiment. "Batch": run GDmicro on all samples altogether.

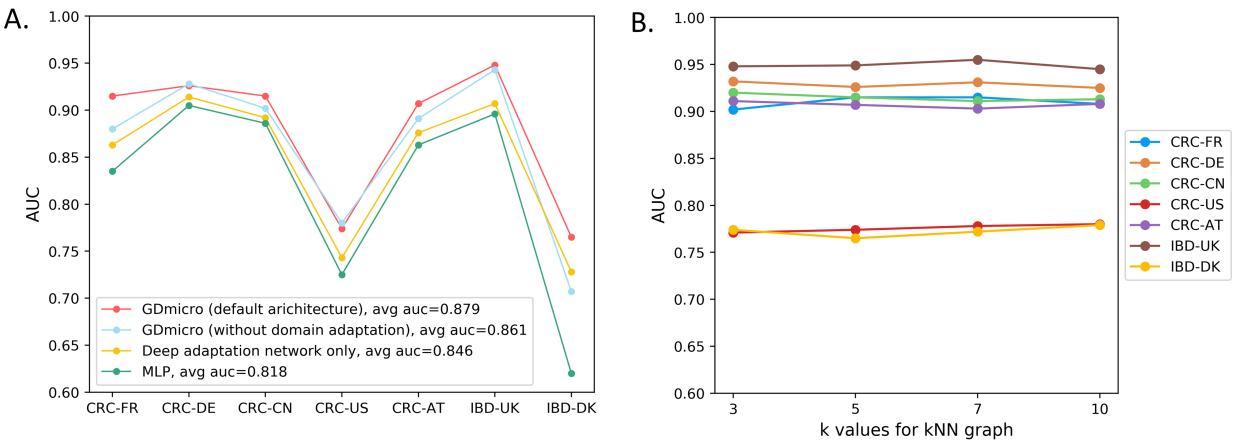

Supplementary Figure S3. (A). The AUC of deep adaptation network, MLP, and GDmicro with different loss functions in LOSO experiments. "avg auc": the average AUC of the tested model in seven cohorts. Deep adaptation network only: applying a single deep adaptation network (Fig. 1 III in main article) to classify host disease status with species abundance data. MLP: applying a multi-layer perceptron to classify host disease status with species abundance data. (B). The AUC of GDmicro with different *k* values in LOSO experiments.

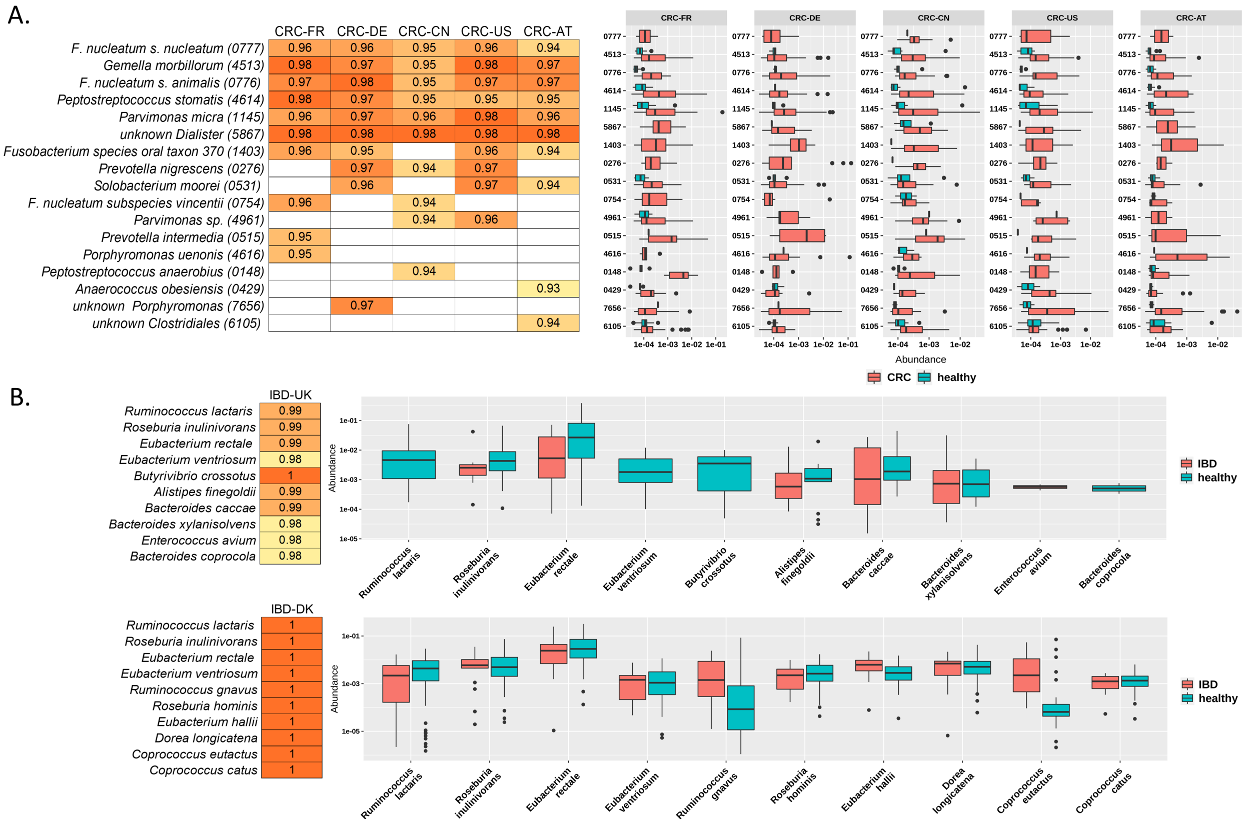

Supplementary Figure S4. (A). The top-10 disease-related species identified by GDmicro on 5 CRC datasets. The values in the cell represent the AUC using that species as the node feature. The boxplot shows the abundance distribution of identified species in CRC patients and healthy samples. (B). The top-10 disease-related species identified by GDmicro on 2 IBD datasets. The values in the cell represent the AUC using that species as the node feature. The boxplot shows the abundance distribution of identified species in IBD patients and healthy samples.

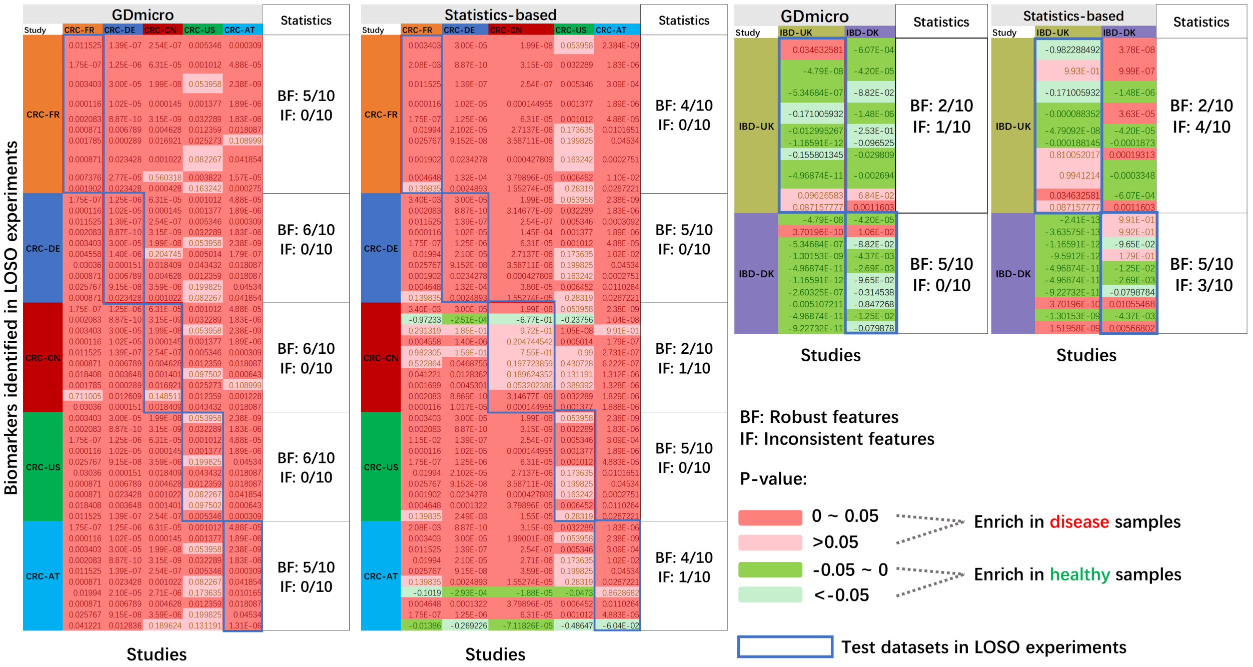

Supplementary Figure S5. P-values of top-10 features identified by GDmicro and statistics-based method in CRC and IBD datasets. Each row represents a feature identified in the test dataset of the LOSO experiment, and each cell corresponds to a p-value of the feature in the specific study. The p-value is calculated using the Wilcoxon test, where a positive value (marked as red) indicates that the feature is enriched in disease samples, while a negative value (marked as green) signifies enrichment in healthy samples. Robust features: these are features exhibiting a consistent positive or negative p-value of less than 0.05 across all tested datasets. Inconsistent features: these are features that display both positive and negative p-values within the tested datasets.

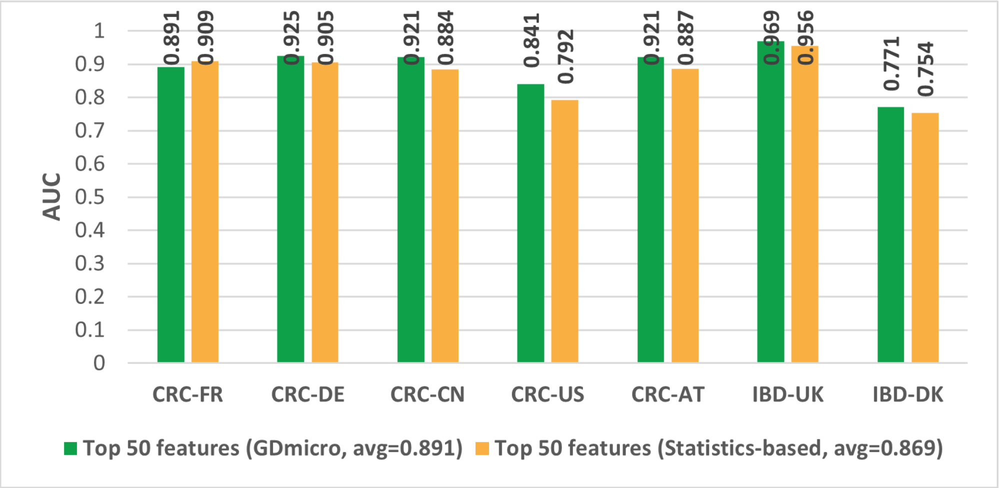

Supplementary Figure S6. The AUC of using the top 50 biomarkers identified by GDmicro and the Wilcoxon test in the LOSO experiment. “avg auc”: the average AUC of the tested method in seven cohorts.

4 Supplementary Tables

Datasets	GDmicro (batch)	GDmicro (single)	SIAMCAT	MetAML	DeepMicro	PopPhy-CNN
CRC-FR	**0.915**	0.853	0.823	0.837	0.806	0.708
CRC-DE	**0.926**	0.88	0.911	0.855	0.699	0.72
CRC-CN	**0.915**	0.876	0.846	0.899	0.649	0.707
CRC-US	**0.774**	0.756	0.71	0.763	0.609	0.723
CRC-AT	**0.907**	0.863	0.847	0.861	0.68	0.781
IBD-UK	**0.949**	0.846	0.504	0.754	0.645	0.783
IBD-DK	**0.765**	0.709	0.689	0.539	0.52	0.611
Average	**0.878**	0.826	0.761	0.786	0.658	0.719

Supplementary Table S1. The cross-study AUC of five tools on seven test cohorts in the LOSO experiment. Bold: the best performance of each cohort.
